# Comments on Julia Moeller’s Paper from a Person-Oriented Perspective

**DOI:** 10.17505/jpor.2025.28093

**Published:** 2025-06-28

**Authors:** András Vargha

**Affiliations:** Institute of Psychology, Károli Gáspár Reformed Church University, Budapest, Hungary, and Institute of Psychology, Eötvös Loránd University, Budapest, Hungary

## Introduction

From a person-oriented perspective Julia Moeller’s (2025) paper on the pitfalls on *z*-standardization has three important take-home messages:

1. Contrary to the long custom followed, it is suggested not to standardize variables before cluster analyses. Raw scores have the advantage that they are easy to understand. Whenever possible, work with the original raw scores.
2. If the variables have different scales, and some transformation is necessary, it is better to create a common scale by means of a POMS transformation than with a *z*-transformation.
3. It is also suggested not to standardize variables after cluster analysis, when we want to use figures to present the results with their cluster means. This implies that in person-oriented analyses not only the analysis itself (whether we standardize the variables or not), but also the way the results are explained or plotted is important.

However, it is an open question how to explain the results of a cluster analysis (CA) on the basis of a plot of cluster means, and how to decide whether a cluster mean can be considered low, average or high, if we do not standardize variables before the CA. In this commentary I will argue that, while accepting the main idea of avoiding *z*-scores before performing a CA, it is very useful to apply a special standardization, called *z_t_*-standardization, on the cluster centroids after the CA has been done, for the sake of obtaining an explainable bar diagram. In *z_t_*-standardization, the cluster means of each variable are transformed with the same linear transformation, using the total mean *M_t_* of the variables (the average of the variable means) and a kind of total *SD*, the square root of the average of the variable variances, *SD_t_*. The big difference between *z*-standardization and *z_t_*-standardization is that in standardizing before CA, each variable *X_j_* is standardized with its own – and generally different – mean and *SD*:

$$Z_{j}=(X_{j}-M_{j})/SD_{j},$$

whereas in *z_t_*-standardization each cluster mean *M_ij_* is standardized with the same common mean (*M_t_*) and *SD* (*SD_t_*):

$$M_{ij}\prime =(M_{ij}-M_{t})/SD_{t},$$

in this way creating a common frame of reference for the different cluster means for the judgements whether they can be considered low, average or high.

As an illustration, I use a male sample of size *N* = 905 from a Hungarian study on substance use during adolescence (Mirnics et al., 2021), analyzed with several CAs by Vargha and Grezsa (2024). The variables of the analyses were the four avoidance and anxiety scales of the mother and father domains of the Close Relationships – Relationship Structures attachment questionnaire (ECR-RS; Fraley et al., 2011), called Mother Avoidance (AvoidMo), Mother Anxiety (AnxMo), Father Avoidance (AvoidFa), and Father Anxiety (AnxFa). These scale values were calculated as the average of the corresponding 5-point items (scores from 1 to 5). Since the original 5-point items of the scales were extremely skewed, yielding highly skewed scales, Vargha and Grezsa (2024) truncated the items (avoidance items to 4, anxiety items to 3), and the attachment scales were then constructed from these truncated items. The resulting value ranges of the two avoidance scales (AvoidMo and AvoidFa) were then [1–4], and of the anxiety scales (AnxMo and AnxFa) [1–3]. The number of usable cases was 793. Comparing different clustering methods, in each case applying *z*-standardization before the analysis, Vargha and Grezsa (2024) found that both standard hierarchical CA and *k*-means CA could discover a 7-type structure, which was also verified by the nonstandard *k*-medians CA.

In the present commentary I will show two methods to create identical measurement scales for the four input variables (AvoidMo, AnxMo, AvoidFa, AnxFa) of CAs, perform *k*-means CAs without *z*-standardization, create bar diagrams based on the *z_t_*-standardized cluster means, and compare the results of the different CAs quantitatively.

### Creating Identical Measurement Scales

The first method to create identical measurement scales was simply to truncate the original 5-point items of AnxMo and AnxFa to the same [1–4] scale as the items of AvoidMo and AvoidFa and to compute the scale values by averaging the corresponding items. This yielded the same measurement scale for the four input variables (Varset1), where the minimum was 1 and the maximum 4.

The second method to create identical measurement scales was applying the POMS transformation suggested by Moeller (2025) performed on the four attachment variables with different measurement scales used in the CAs of Vargha and Grezsa (2024). This yielded again the same measurement scale for the four input variables (Varset2), where the minimum was 0 and the maximum 1. The POMS transformation of a value *x* of a variable *X* is as follows:

$$\text{POMS}(x)=(x-x_{min})/(x_{max}-x_{min}),$$.

where *x_min_* and *x_max_* are the smallest and largest *X* values in the sample (Cohen et al., 1999; Gower, 1971).

### *k*-Means Cluster Analyses Without *z*-Standardization

**Figure 1:**
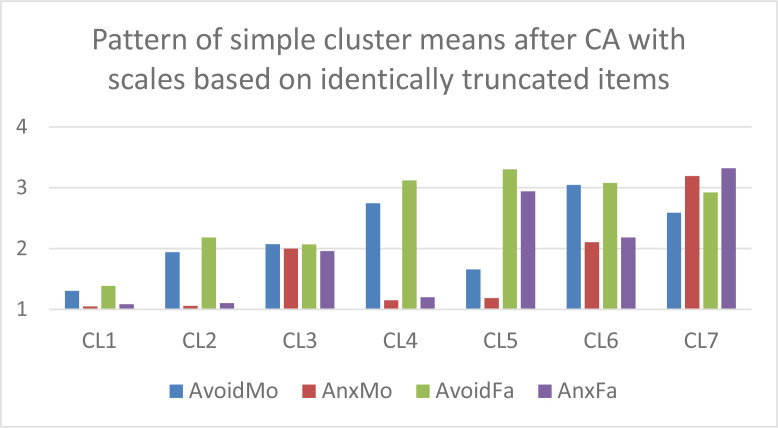
The pattern of the 7-cluster k-means solution of Varset1 variables (clusters arranged in descending order of overall attachment level).

**Figure 2:**
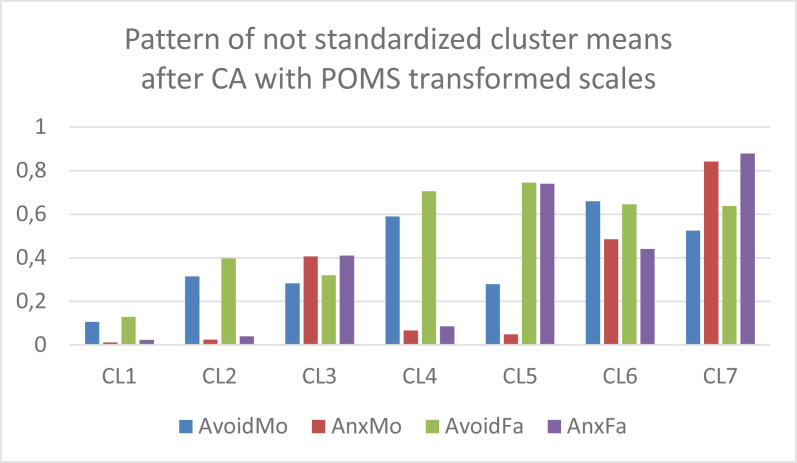
The pattern of the 7-cluster k-means solution of Varset2 variables (clusters arranged in descending order of overall attachment level).

**Figure 3:**
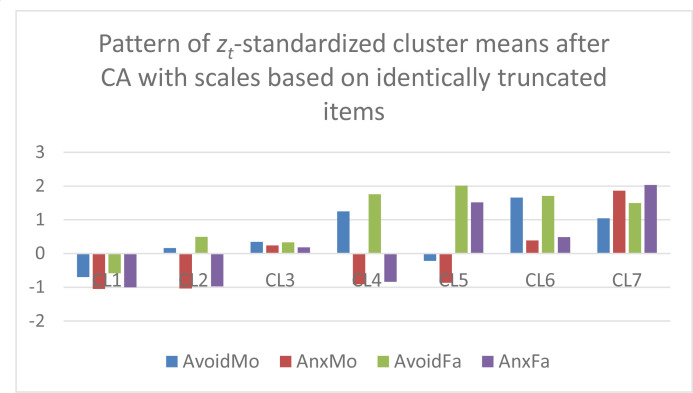
The z_t_-standardized centroid pattern of the 7-cluster k-means solution of Varset1 variables (clusters arranged in descending order of overall attachment level).

**Figure 4:**
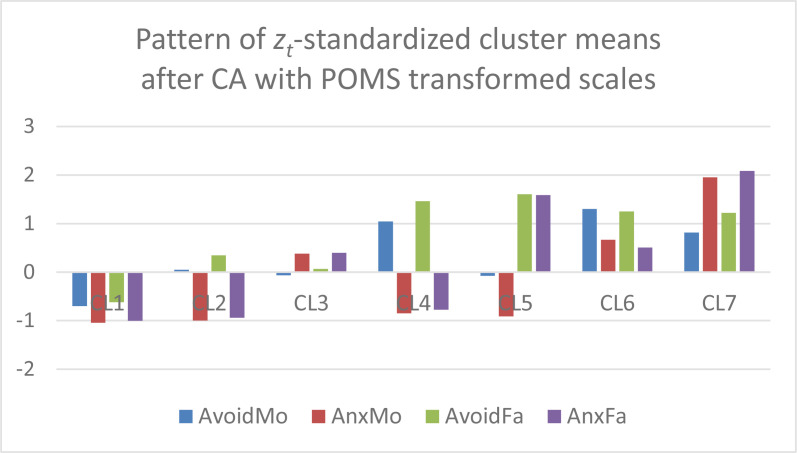
The z_t_-standardized centroid pattern of the 7-cluster k-means solution of Varset2 variables (clusters arranged in descending order of overall attachment level)

In the next step, *k*-means CAs were performed without *z*-standardization and with *k* = 7 clusters on the Varset1 and Varset2 sets of input variables, respectively. The analyses were performed using the latest versions of ROP-R (Vargha & Bánsági, 2022; Vargha & Grezsa, 2024) and ROPstat (Vargha et al., 2015) that provide the tables of raw cluster means and *z_t_*-standardized cluster means. Based on these tables, bar diagrams of the cluster centroids were created in Excel for both the Varset1 and the Varset2 variables. In Figures 1 and 2 we can see the patterns of raw cluster centroids, and in Figures 3 and 4 the patterns of *z_t_* -standardized cluster centroids.

Based on Figures 1 to 4 we can draw the following conclusions.

1. CAs with Varset1 and Varset2 revealed very similar cluster structures.
2. Figures created with the raw cluster means (Figures 1 and 2) clearly show the differences between the seven cluster centroids but are not suitable for drawing conclusions about which mean is considered low, average or high.
3. However, figures created with the *z_t_*-standardized cluster means (Figures 3 and 4) are perfectly suitable for this purpose. For example, CL1 is the cluster of overall good parental attachment where both avoidance and anxiety are at low levels, CL7 is the cluster of overall bad parental attachment, where both avoidance and anxiety are at high levels (AnxMo and AnxFa are at very high levels), CL4 is the cluster, where parental avoidance is at high level, but parental anxiety is at low level, etc.

**Figure 5:**
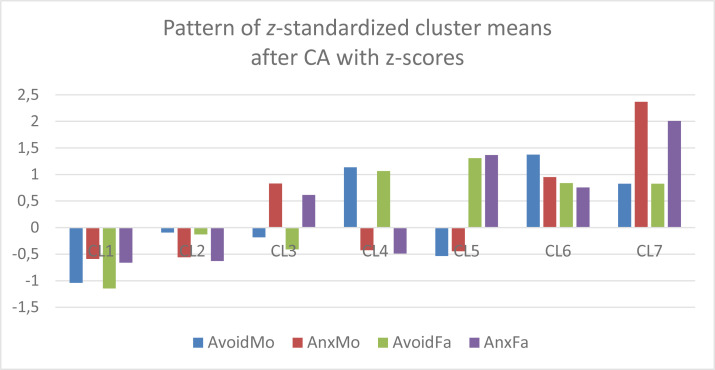
The z-standardized centroid pattern of the 7-cluster k-means solution of the original four variables based on differently truncated items (clusters arranged in ascending order of overall attachment level).

In order to make a comparison between the 7-cluster *k*-means solution obtained in Vargha and Grezsa (2024) carried out on *z*-scores of the original four attachment variables, the z-standardized centroid pattern of this solution was plotted in Figure 5. Comparing Figures 3 and 4 with Figure 5 we find similarities, but it is also evident that some clusters of the CA solution of *z*-scores (e.g., CL2, CL5 and CL6) differ markedly from the corresponding clusters of the Varset1 and Varset2 solutions.

### Quantitative Comparisons of the Results of the Different Cluster Analyses

Table 1 contains some quality coefficients (see Vargha et al., 2016) for the cluster solutions of the three different CAs plotted on Figures 3, 4 and 5. The adequacy of these solutions seems to be very similar, with a small superiority of Varset2, the POMS transformed scales in terms of cluster homogeneity (EESS% is the largest, HCmeanS is the smallest).

**Table 1:** Some quality coefficients for different cluster solutions.

Input variables with the code of the obtained CA solution	EESS%	PB	XBmod	SC	HCmeanS	HCstan minmax
1. Scales based on differently truncated items: k7orig	78.5	0.418	0.554	0.672	0.435	0.20–0.94
2. Scales based on identically truncated items (Varset1): k7id	77.5	0.400	0.528	0.667	0.455	0.20–1.10
3. POMS transformed scores of variables of k7orig (Varset2): k7P	80.0	0.404	0.471	0.675	0.404	0.15–0.93

Note. EESS% = Explained error sum of square percentage; PB = cluster point biserial correlation; XBmod = Modified Xie-Beni index; SC = Simplified Silhouette coefficient; HCmeanS = average *z_t_*-standardized within cluster difference.

The similarity of the three different cluster structures can be assessed by means of pairwise frequency tables, and the cell matching ratios computed based on these frequency tables. The cell matching ratio of two clusters of different solutions is the cell frequency divided by the harmonic mean of the corresponding row and column totals. As Table 2 shows, the seven clusters of the three CAs were very similar, with the largest similarities between the corresponding CL1, CL2 and CL4 clusters. CL1 represents the type of best parental attachment, CL2 a good parental attachment with an average level of parental avoidance and a very low level of parental anxiety, and CL4 a nontrivial type with a high level of parental avoidance and a low level of parental anxiety.

**Table 2:** Cell matching ratios for each cluster for the three pairs of cluster solutions.

Pair of CA solutions	CL1	CL2	CL3	CL4	CL5	CL6	CL7
k7orig, k7id	.98	.97	.82	.94	.88	.80	.84
k7orig, k7P	.98	.98	.92	.97	.85	.94	.96
k7id, k7P	.97	.97	.76	.95	.83	.75	.82

Finally, the pattern of *z_t_*-standardized means in the 7-cluster *k*-means solution of POMS transformed Varset2 variables are summarized in Table 3 with the cluster sizes (CLsize) and the standardized cluster homogeneity coefficients (HCstan). From here we see that the most stable clusters across the three CAs (namely CL1, CL2 and CL4) are the largest and the most homogeneous, with the smallest HCstan values. In this solution 64.7% of the total sample belongs to a cluster with a HCstan value less than .50. Since this proportion is less than 54% for the other two cluster solutions, the CA with the POMS transformed input variables can be regarded as the most successful.

**Table 3:** Pattern of z_t_-standardized means in the 7-cluster k-means solution of Varset2 variables (L = Low, H = High; more pluses indicate more extreme means).

Cluster	AvoidMo	AnxMo	AvoidFa	AnxFa	CLsize	HCstan
CL1	L	L+	(L)	L+	200	0.15
CL2	.	L	.	L	209	0.21
CL3	.	.	.	.	83	0.52
CL4	H+	L	H++	L	104	0.48
CL5	.	L	H++	H++	52	0.93
CL6	H+	(H)	H+	(H)	67	0.78
CL7	H	H+++	H+	H++++	78	0.68

### Summary

In summary, we can conclude that it is indeed good advice not to standardize variables separately and differently before cluster analyses. With such a transformation, the original meaning of the scale units is lost, and when calculating distances, the different variables will be weighed in proportion to the inverse of their variance. However, a special *z*-standardization, called *z_t_*-standardization of the cluster means, using the total mean *M_t_* of the variables and the *SD_t_*, the square root of the pooled common variance after a cluster analysis, seems to be useful for making explainable bar diagrams.

If the variables have different units of measurement, it is necessary to bring them to the same scale. The POMS transformation (Cohen et al., 1999) seems to be appropriate for generating common scales. However, sometimes a simple linear transformation can be used to bring the input variables to a common scale. To achieve this, it is useful, for example, to construct scales of questionnaires with different numbers of items by averaging the items in the scale (and not summing the items), which preserves the common range of values of the items. And if, for example, a scale *X* of questionnaire A has a range of values [1-4] and a scale *Y* of a questionnaire B has a range of values [1-7], making a common scale can be achieved in the following way. First, we subtract 1 from both *X* and *Y*, obtaining a scale for *X* [0-3] and for *Y* [0-6]. We see that the range of *Y* is twice as wide (6/3 = 2), so we multiply *X* by 2, having this way for *X* the value range [0-6], the same as for *Y*.

## Data Availability

The research data analyzed in this paper are available from the author on request.

## Action editor

Lars-Gunnar Lundh served as action editor for this article.

## Open access

This article is distributed under the terms of the Creative Commons Attribution 4.0 International License (http://creativecommons.org/licenses/by/4.0/), which permits unrestricted use, distribution, and reproduction in any medium, provided you give appropriate credit to the original author(s) and the source, provide a link to the Creative Commons license, and indicate if changes were made.
